# MYB80 homologues in Arabidopsis, cotton and Brassica: regulation and functional conservation in tapetal and pollen development

**DOI:** 10.1186/s12870-014-0278-3

**Published:** 2014-10-14

**Authors:** Yue Xu, Sylvana Iacuone, Song Feng Li, Roger W Parish

**Affiliations:** Botany Department, La Trobe University, AgriBio Centre, Melbourne, Victoria 3086 Australia

**Keywords:** Brassica, Cotton, *Gossypium hirsutum*, Male sterility, *MYB80*, Transcription factor

## Abstract

**Background:**

The Arabidopsis AtMYB80 transcription factor regulates genes involved in pollen development and controls the timing of tapetal programmed cell death (PCD). Downregulation of *AtMYB80* expression precedes tapetal degradation. Inhibition of *AtMYB80* expression results in complete male sterility. Full-length *AtMYB80* homologs have been isolated in wheat, rice, barley and canola (C genome).

**Results:**

The complete sequences of *MYB80* genes from the *Brassica. napus* (A gene), *B. juncea* (A gene), *B. oleracea* (C gene) and the two orthologs from cotton (*Gossypium hirsutum*) were determined. The deduced amino acid sequences possess a highly conserved MYB domain, 44-amino acid region and 18-amino acid C-terminal sequence. The cotton MYB80 protein can fully restore fertility of the *atmyb80* mutant, while removal of the 44 amino acid sequence abolishes its function. Two conserved MYB *cis*-elements in the *AtMYB80* promoter are required for downregulation of *MYB80* expression in anthers, apparently via negative auto-regulation. In cotton, tapetal degradation occurs at a slightly earlier stage of anther development than in Arabidopsis, consistent with an earlier increase and subsequent downregulation in *GhMYB80* expression. The MYB80 homologs fused with the EAR repressor motif have been shown to induce male sterility in Arabidopsis. Constructs were designed to maximize the level of male sterility.

**Conclusions:**

*MYB80* genes are conserved in structure and function in all monocot and dicot species so far examined. Expression patterns of *MYB80* in these species are also highly similar. The reversible male sterility system developed in Arabidopsis by manipulating *MYB80* expression should be applicable to all major crops.

**Electronic supplementary material:**

The online version of this article (doi:10.1186/s12870-014-0278-3) contains supplementary material, which is available to authorized users.

## Background

The *AtMYB80* transcription factor is involved in tapetum and pollen development and is required for the regulation of tapetal programmed cell death (PCD) in developing Arabidopsis anthers [1-3]. Using 3.2 kb of the *AtMYB80* promoter fused to the *GUS* reporter gene and *in-situ* hybridization analysis, expression of *AtMYB80* was found in the tapetum, middle layers and developing microspores from anther developmental stages 5 to 9 [1,4]. Functional disruption of *AtMYB80* results in complete male sterility with early tapetum degeneration and collapsed pollen [2,4,5]. Three genes directly regulated by AtMYB80 have been identified using ChIP analysis, namely an A1 aspartic protease (*UNDEAD*), a pectin methylesterase (*VANGUARD1*) and a glyoxal oxidase (*GLOX1*). Premature tapetal PCD and degeneration were observed in the *undead* and *atmyb80* mutants [3].

The *AtMYB80* homologs from rice (*Oryza sativa*), wheat (*Triticum aestivum*), barley (*Hordeum vulgare*) and canola (*Brassica napus*) have been isolated and their protein sequences show significant conservation [6]. High similarity occurs between the R2R3 MYB domains, the 44-amino acid region immediately downstream of the MYB domain and an 18-amino acid sequence at the C-terminus [2,6]. The expression patterns driven by the *OsMYB80*, *TaMYB80* and *BnMYB80* promoters in Arabidopsis are similar to that of *AtMYB80*, being restricted to the tapetum and developing microspores and occurring from stages 6 to 10. When driven by the *AtMYB80* or their native promoters, the full-length *OsMYB80*, *TaMYB80* and *BnMYB80* constructs are able to fully restore the fertility of the male sterile *atmyb80* T-DNA mutant [6].

The two agriculturally important oilseed Brassica species, canola (*B. napus*, genome AACC) and brown mustard (*B. juncea*, genome AABB), originate from hybridisation between pairs of the diploid species *B. rapa* (AA), *B. nigra* (BB), and *B. oleracea* (CC) [7,8]. The full-length *BnMYB80* of the C genome has been isolated [6], while the *MYB80* orthologs from the A genome of *B. napus* and *B. juncea* and C genome of *B. oleracea* have not yet been identified. Upland cotton (*Gossypium hirsutum* L., genome A^T^D^T^) is the most widely cultivated allotetraploid species and originated from interspecific hybridization between *G. arboreum* (genome A^1^) and *G. raimondii* (genome D^5^) [9]. Only one MYB transcription factor, *GhMYB24*, has so far been found to play a role in cotton anther development [10]. *GhMYB80* is the cotton homolog of *AtMYB80*. Two partial coding sequences of *GhMYB80* were separately obtained and the deduced amino acid sequence shares high similarity with MYB80 homologs in other species [6]. However, the full-length DNA sequence of each *GhMYB80* ortholog is still lacking. The expression pattern of *GhMYB80* has not been determined and whether functional conservation exists between AtMYB80 and GhMYB80 is unknown.

The utilization of cytoplasmic male sterility (CMS) and nuclear encoded fertility restore genes (*Rf*) is an important technology for hybrid cotton and canola production [11,12]. However, the CMS-based hybridization system is difficult to develop and maintain [13]. Furthermore, the CMS phenotype is often unstable under both high and low temperatures [14-16]. Manipulation of expression of the *MYB80* transcription factor provides a novel means to induce and subsequently reverse male sterility, facilitating the production of hybrid plants [2]. The experiments described here were aimed at cloning the *MYB80* genes from cotton and Brassica (A and C genomes) and comparing their protein structures and promoter sequences. The expression pattern of the *GhMYB80* gene in cotton anthers and its capacity to rescue the male sterile *atmyb80* mutant were determined. The role of a conserved 44 amino acid sequence in MYB80 function was further assessed. The effectiveness of GhMYB80 and BnMYB80 proteins to induce male sterility in Arabidopsis was examined, when fused to the EAR sequences.

## Results

### Cloning of the homologous *MYB80* genes from Brassica and cotton

**Figure 1:**
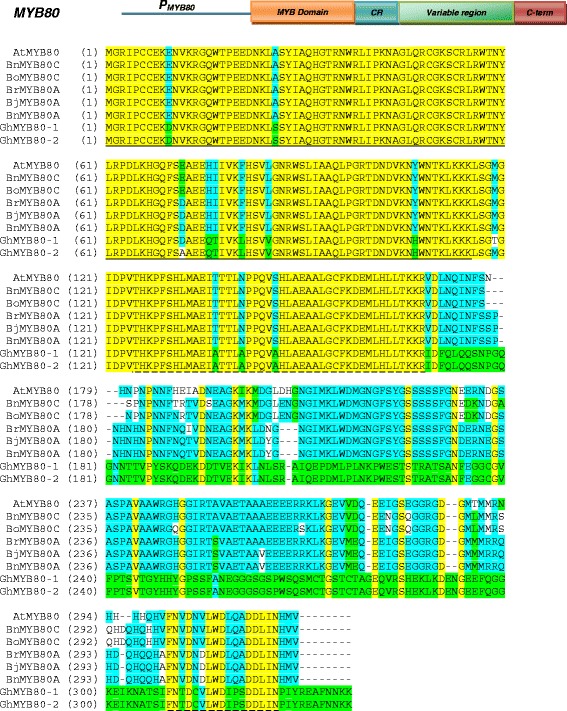
**Diagram of the sequence alignment of the homologous MYB80 proteins.** Sequences include AtMYB80 (*A. thaliana*), BnMYB80 (*B. napus*), BrMYB80 (*B. rapa*), BjMYB80 (*B. juncea*), BoMYB80 (*B. oleracea*) and GhMYB80 (*G. hirsutum*). Yellow highlight represents the conserved amino acids between all the homologs. Blue and green highlight represents the conserved amino acids between the Brassica and cotton MYB80 homologs, respectively. The underline indicates the MYB domains and the dash lines indicate the two conserved regions in the C-termini. CR, conserved region; C-term, C-terminus.

The homologous *MYB80* genes from *B. napus* (A gene), *B. juncea* (A gene), *B. oleracea* (C gene) and *G. hirsutum* were cloned and sequenced. The nucleotide sequences and the deduced amino acid sequences were compared with Arabidopsis *AtMYB80* [1], *B. napus* MYB80 (C gene) [6] and *B. rapa MYB80* (A gene) obtained from the GenBank (GI: 110797058) (Figure 1 and Additional file 1: Figure S1). The nucleotide sequences of the eight *MYB80* homologs are highly conserved in their exons. The amino acid sequences are highly similar in the MYB domain (amino acids 1 – 115), a 44-amino acid region adjacent to the MYB domain (amino acids 125 – 168), and a 18 amino acid region at the end of the C-termini. A variable region of 131 to 139 amino acids is present between the 44-amino acid and the C-terminal sequences, sharing 10.7% identity (Figure 1). Among the five MYB80 homologs of the Brassica species, the amino acid sequences in the variable region of the three A genes are more similar to each other than that of the two C genes (99.1% vs. 97.8% identity). The *MYB80* homolog of the Brassica B gene has not yet been cloned. The two *MYB80* ortholog genes (*GhMYB80-1* and *2*) from *G. hirsutum* are highly conserved, sharing 98.4% and 99.4% identity in their nucleotide and peptide sequences, respectively (Figure 1 and Additional file 1: Figure S1). The two genes are likely to be derived from the A and D genomes.

### Deletion and mutagenesis analysis of the *AtMYB80* promoter

**Figure 2:**
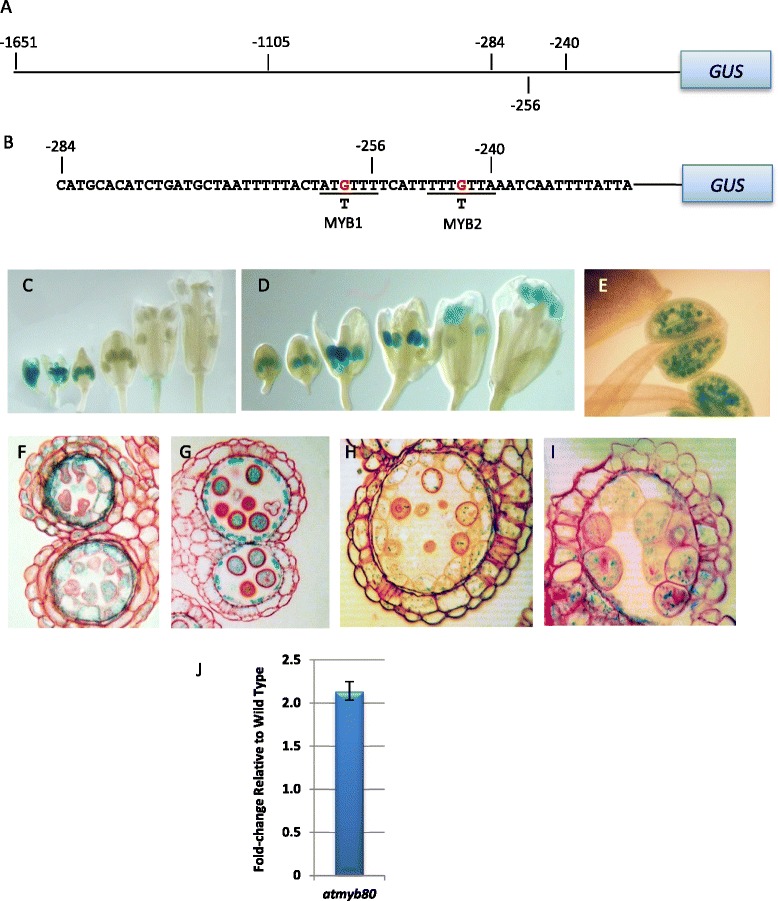
**Autoregulation of the** ***AtMYB80*** **promoter. A**. A schematic diagram of *AtMYB80* promoter-*GUS* deletion constructs. Numbers indicates the length of *AtMYB80* promoter used for each construct. **B**. A schematic diagram of mutagenesis constructs within the −284 to -240bp *AtMYB80* promoter region. Nucleotides that were targeted for mutagenesis are in red with the corresponding change indicated directly below. **C**. Floral bud line-up (stages 7 to 12) of the control line showed GUS activity was extended until stage 9. **D**. Floral bud line-up (stages 7 to 12) of the *M2* line showed GUS activity extended to stage 12. **E**. GUS activity was present in the *M2* anther at stage 12. **F** and **G**. Cross-sections of *M2* anthers showed GUS activity in the tapetum, the outer tapetal cell wall and developing microspores at stages 9 **(F)** and 11 **(G)**. **H** and **I**. GUS activities were present in the tapetum and collapsing pollen grains of the homozygous *atmyb80* mutant possessing a wild-type *AtMYB80* promoter-*GUS* construct at stage 10 **(H)** and 12 **(I)**. **J**. Comparative qRT-PCR analysis of *AtMYB80* transcript levels in the young floral buds (anther stages 5 to 9) of the *atmyb80* mutant versus wild-type. The *AtMYB80* transcript level is higher in the *atmyb80* mutant young floral buds. The *UBQ10* was used as the reference gene. Error bar represents SD.

To delineate the region of the *AtMYB80* 5’UTR/promoter responsible for directing expression to the tapetum and pollen, a series of four *AtMYB80* promoter-*GUS* deletion constructs were prepared. These constructs incorporated 1651, 284, 256 or 240bp of the *AtMYB80* 5’UTR sequence (relative to the ATG translational start codon) into the pBI vector and were transformed into the wild-type Arabidopsis (Figure 2A). The histochemical GUS staining of florets from the transgenic lines was compared to that of the pPG construct possessing a 3200bp *AtMYB80* promoter [1]. Similar GUS intensity was present in the young florets with the 3200 and 1651bp promoters. No GUS activity was detected in the *240-pBI* transgenic lines. When compared with the *1651-pBI* lines, very weak and weak/moderate GUS intensity was present in the *256-pBI* and *284-pBI* lines, respectively (Additional file 2: Table S1).

The −284 to -240bp sequence of the *AtMYB80* promoter possesses two putative *cis*-elements, namely MYB1 and MYB2. When the MYB1 element was mutated in a 1105bp promoter (construct *M1*, single base change, Figure 2B), *GUS* expression in the anther was unaffected (Figure 2C). However, when MYB1 and MYB2 elements were both mutated (construct *M2*, Figure 2B), GUS activity persisted through to stage 12 (Figure 2D) rather than being downregulated at stage 10. The activity at stage 11 was localized in the microspores or degenerating tapetal layer (Figure 2G). Pollen grains in stage 12 anthers also expressed GUS activity (Figure 2E). Both the MYB1 and MYB2 elements of the *AtMYB80* promoter are conserved in the C genome of Brassica but not in the other four *MYB80* genes. MYB2 is conserved in the *GhMYB80* promoter and MYB1 in the *BnMYB80* A gene promoter (Additional file 3: Figure S3).

To examine whether the expression of *AtMYB80* is auto-regulated, a promoter-*GUS* construct possessing a 1105bp *AtMYB80* promoter was introduced into an *atmyb80* T-DNA insertion mutant (Figure 2A). Homozygous *atmyb80* plants are completely male sterile whilst heterozygous plants are fully male fertile [2]. GUS activity was observed in the anthers of the heterozygous *atmyb80* mutant from stages 5 to 9, the same as previously described [1,2]. *GUS* expression was extended to stage 13 in the two homozygous *atmyb80* mutant lines (Additional file 4: Table S2). GUS activity was present in the largely vacuolated tapetal layer (stage 10) and collapsing pollen grains (stage 12) (Figure 2H and I).

Transcript levels of *AtMYB80* in both wild-type and *atmyb80* mutant were analysed using real-time qRT-PCR. The level of truncated *AtMYB80* transcript was approximately 2.1 fold higher in the young mutant floral buds (anther developmental stages 5 to 9) than that of the wild-type (Figure 2J). Previous microarray data comparing differential gene expression in the wild-type and *atmyb80* mutant anthers showed a 3.2 fold (p value 0.012) up-regulation of the truncated *AtMYB80* transcript in the mutant (unpublished data) [3]. These results together suggest *AtMYB80* is involved in the negative auto-regulation.

The promoters of all eight *MYB80* genes possess a highly conserved sequence approximately −300 to -380bp upstream of the ATG codon. Four *cis*-elements are conserved in all six genes, including W-box (TTGAC), MYB (A/TACC), GTGANTG10 (TCAC) and DOFCOREZM elements (A/TAAAG) (Additional file 1: Figure S1).

### *GUS* expression driven by the *GhMYB80* promoter in Arabidopsis

**Figure 3:**
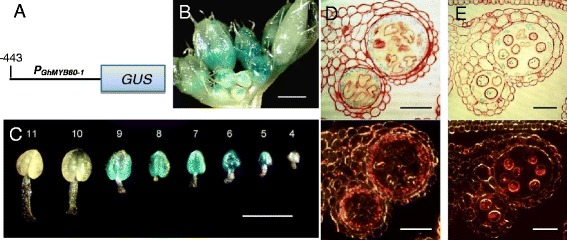
**Analysis of the spatial and temporal expression pattern driven by the** ***GhMYB80-1*** **promoter in Arabidopsis. A**. A schematic diagram of the *GhMYB80-1* promoter-*GUS* construct. **B**. GUS activity is detected in developing *P*_*GhMYB80*_ *-GUS* floral buds. **C**. Line-up (anther stages 4 to 11) of the *P*_*GhMYB80*_ *:GUS* anther after GUS staining. **D** and **E**. Sections of the *P*_*GhMYB80*_ *:GUS* anthers stained with safranin. Light and dark-field microscopy of stage 8 **(D)** and stage 9 **(E)** anthers. Bars = 500 μm in **B** and **C**. Bars = 25 μm in **D** and **E**.

To ascertain whether the *GhMYB80* promoter resembles the *AtMYB80* promoter in driving expression in the Arabidopsis anther, the *GUS* reporter gene was used. The *GhMYB80-1* promoter employed was 443 bp in length (numbered from the ATG). An anther line up showed GUS activity first appeared at stage 5 and persisted to stage 9 (Figure 3C). No activity was detected at stages 10 and 11. Light and dark field microscopy of anther sections showed GUS activity in the tapetum and microspores at stages 8 and 9 (Figure 3D and E). Hence, the expression pattern driven by the *GhMYB80-1* promoter in Arabidopsis resembles that of the *AtMYB80* promoter.

### Transcript levels of *GhMYB80* in developing cotton anthers

**Figure 4:**
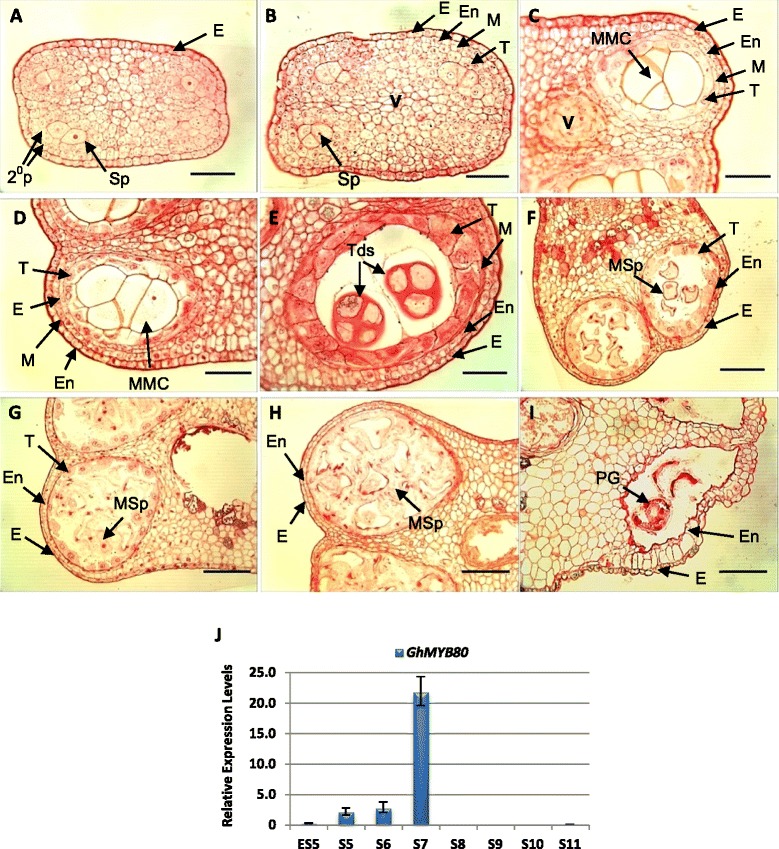
**Semi-thin sections of developing** ***G. hirsutum*** **anthers and relative transcript levels of** ***GhMYB80*** **in anthers.** The indicative bud sizes for each anther developmental stage were measured (Additional file 5: Table S3). **A**. At stage 3, the secondary parietal layers and sporogenous cells are apparent. **B**. At stage 4, formation of the epidermis, endothecium, middle layer and tapetum has been initiated. **C**. At stage 5, the microspore mother cells appear. **D**. At stage 6, the microspore mother cells commence meiosis and the tapetal cells become vacuolated. **E**. At stage 7, the tapetal cytoplasm is condensed and tetrads appear in the anther locules. **F**. At late stage 8, microspores are released from the tetrads. Tapetal cell walls have been degraded. **G**. At stage 9, the tapetum degeneration appears to commence. Microspores are vacuolated. **H**. At stage 10, the tapetum has been degraded. Remnants of tapetal cells are visible. The microspores are still vacuolated. **I**. At stage 11, early pollen grains appear. 2°P, secondary parietal layer; **E**, epidermis; En, endothecium; MSp, microspores; ML, middle layer; MMC, microspore mother cell; MSp, microspore; PG, pollen grains; Sp, sporogenous cells; T, tapetum; Tds, tetrads; V, vascular. Scale bars = 50 μm in **A**, **B**, **C**, **D** and **E**. Scale bars = 100 μm in **F**, **G**, **H** and **I**. **J**. Relative expression levels of the *GhMYB80* in the wild-type *Gossypium hirsutum* anther. The *GhMYB80* transcription level was relatively low at early stage 5 (ES5), stages 5 and 6. It reached a peak level at stage 7 of anther development and was absent from late stage 8 (LS8) to stage 11. The *G. hirsutum UBIQUITIN* (*UBI1*) was used as the reference gene. S5 to S11, stages 5 to 11. Error bar represents SD.

The indicative sizes (length and width) of cotton floral buds corresponding to anther developmental stages were determined using semi-thin sections (Additional file 5: Table S3). The anther stages (from 3 to 11) were numbered in accordance with the morphological changes used for defining the stages of Arabidopsis anther development [17,18]. At stage 4, formation of the tapetum in cotton anthers was initiated (Figure 4B). At stage 6, the tapetal layer became vacuolated (Figure 4D). The tapetal cytoplasm was condensed at stage 7 (Figure 4E) and cell walls degraded at stage 8 (Figure 4F). Tapetal cell degeneration appeared to commence at stage 9 (Figure 4G) and tapetal layer was no longer visible at stage 10 (Figure 4H). The transcript levels of *GhMYB80* in cotton anthers at the developmental stages 5 to 11 were analysed using real-time qPCR (Figure 4J) and RT-PCR (Additional file 6: Figure S2). The *GhMYB80* transcript level was very low at early stage 5, subsequently increasing at stages 5, 6 and 7. The major increase was from stage 6 to 7 when the tapetal cytoplasm becomes condensed and tetrads appear. At late stage 8, *GhMYB80* transcripts could no longer be detected.

### GhMYB80 can rescue the male sterile Arabidopsis *atmyb80* T-DNA mutant

**Figure 5:**
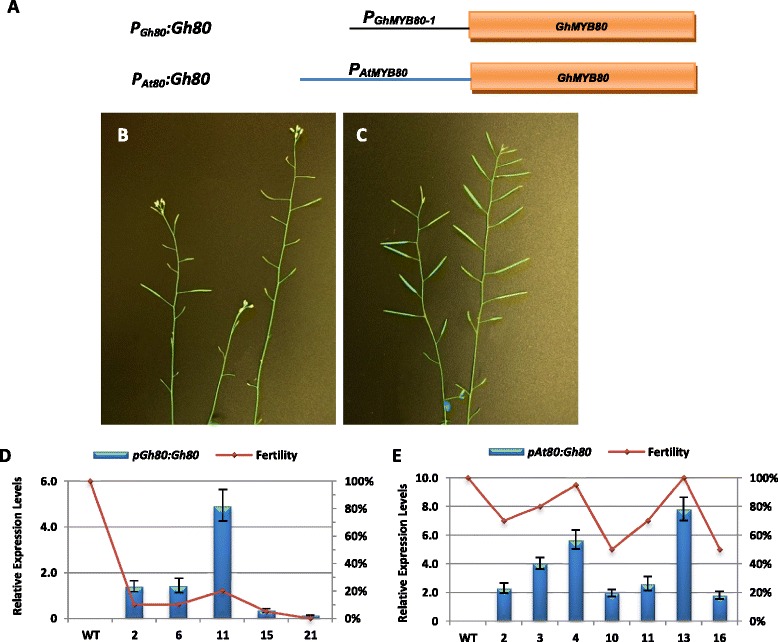
**Silique phenotype and expressional analyses of transgenes in the** ***P***_***Gh80***_ ***:Gh80*** **and** ***P***_***At80***_ ***:Gh80*** **Arabidopsis lines. A**. Schematic representation of the *P*_*Gh80*_ *:Gh80* and *P*_*At80*_ *:Gh80* complementation constructs. **B**. **A** *P*_*Gh80*_ *:Gh80* transformed *atmyb80* homozygous mutant (line 11) exhibiting 20% fertility. **C**. **A** *P*_*At80*_ *:Gh80* transformed *atmyb80* homozygous mutant exhibiting 100% fertility (line 13). **D** and **E**. The transcript levels of *P*_*Gh80*_ *:Gh80* **(D)** and *P*_*At80*_ *:Gh80* **(E)** relative to the *UBQ10* reference gene are positively correlated with plant fertility in the selected lines. Wild type (WT) is the negative control. Error bar represents SD.

To determine whether the GhMYB80 and AtMYB80 are functionally conserved, the *atmyb80* mutant was transformed with the full-length *GhMYB80-1* coding sequence under the control of its own promoter (443bp; *P*_*Gh80*_*:Gh80*) or the *AtMYB80* promoter (1100bp; *P*_*At80*_*:Gh80*) (Figure 5A). The homozygous *atmyb80* T-DNA insertion mutants possessing the transgenes were identified using PCR. Plant fertility is defined as the percentage of the elongated siliques versus the total siliques. In one of the ten *P*_*Gh80*_*:Gh80* transformed *atmyb80* homozygous mutants, fertility was partially restored (20% fertility) (Figure 5B). The other nine lines were less than 10% fertile or remained completely sterile. However, fertility of the nine *atmyb80* homozygous lines carrying the *P*_*At80*_*:Gh80* transgene was significantly or fully restored, resulting in 50-100% fertility (Figure 5C). The expression levels of the *P*_*Gh80*_*:Gh80* and *P*_*At80*_*:Gh80* genes in the relevant transgenic lines were determined using real-time quantitative PCR. Plant fertility was positively correlated with the relative expression levels of the transgenes (Figure 5D and E). The *GhMYB80-1* promoter is apparently not as effective as the *AtMYB80* promoter in Arabidopsis.

### The effects of removing the 44-amino acid or the C-terminal region on MYB80 activity

**Figure 6:**
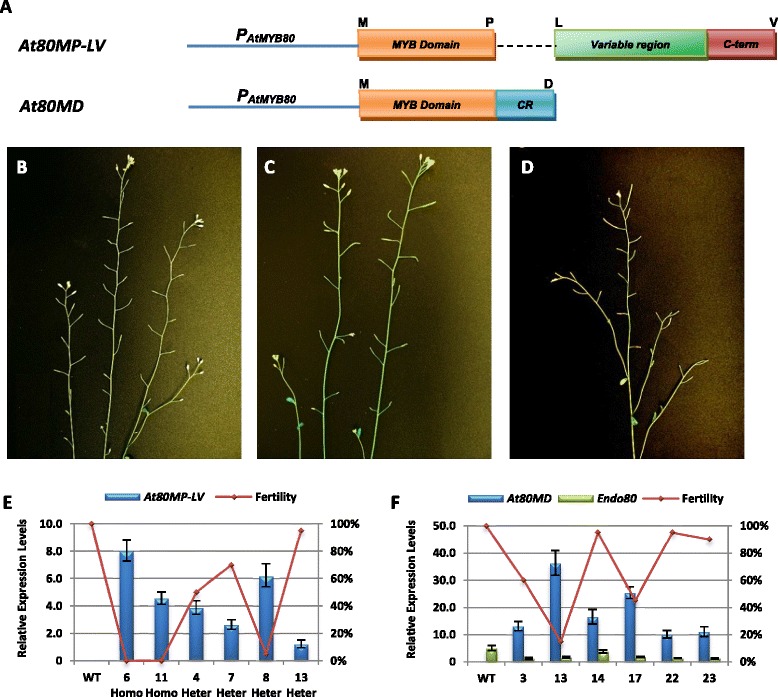
**Silique elongation and expressional analyses of transgenes in the** ***At80MP-LV*** **and** ***At80MD*** **Arabidopsis. A**. A schematic representation of the *At80MP-LV* and *At80MD* truncated constructs. The letters indicate amino acids at the beginning and end of domains. **B**. The *At80MP-LV* transgene was unable to rescue the *atmyb80* homozygous mutant and the plant remained completely male sterile (line 6). **C**. Plant fertility was reduced in the heterozygous *atmyb80* mutant transformed with the *At80MP-LV* transgene (line 8). **D**. The partially male sterile phenotype of the wild-type Arabidopsis transformed with the *At80MD* construct (line 13). **E**. The expression of *At80MP-LV* was detected in the homozygous *atmyb80* mutants (line 6 and 11, 0% fertility) and the heterozygous *atmyb80* mutants (line 4, 7, 8 and 13, 5% to 95% fertility). **F**. *At80MD* transcript levels and plant fertility were determined in the selected lines. The expression levels of endogenous *AtMYB80* were reduced in all lines. Wild type (WT) is the negative control. Error bar represents SD.

To examine the functions of the 44-amino acid region and the C-terminus of MYB80 protein, two truncation constructs were created by either removing the 44-amino acid region (*At80MP-LV*) or the variable region and C-terminus (*At80MD*) from the protein (Figure 6A). The *At80MP-LV* construct was introduced into the *atmyb80* mutant and the *At80MD* construct transformed into wild type Arabidopsis. Silique elongation and pollen viability were examined in the transgenic lines. All twelve *atmyb80* homozygous lines transformed with the *At80MP-LV* transgene failed to elongate siliques (0% fertility) (Figure 6B). Hence, the 44-amino acid domain is essential for MYB80 activity and may be required for the binding of the R2R3 MYB domain to *cis*-elements in the promoter of target genes. A wide variation in fertility (from 5% to 95%) was found in the *At80MP-LV* transgenic *atmyb80* heterozygous lines (Figure 6C). qRT-PCR examined expression of the *At80MP-LV* transgene in two *atmyb80* homozygous and four heterozygous lines. Severe male sterility (5% fertility) was observed in line 8 where a high level of the *At80MP-LV* expression was detected (Figure 6E). In the heterozygous lines, the At80MP-LV protein may be competing for proteins that bind to the C-terminus of endogenous AtMYB80 and are required for MYB80 activity.

**Figure 7:**
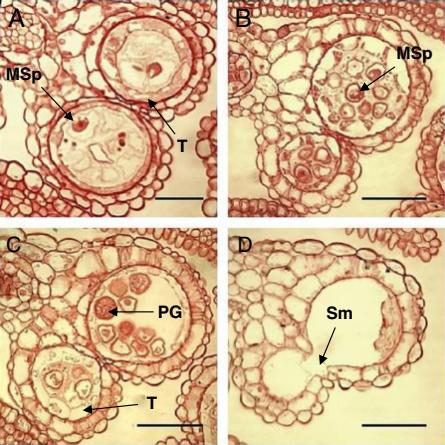
**Semi-thin sections of developing Arabidopsis anthers from a transgenic** ***At80MD*** **plant (line 13). A**. Stage 8; vacuolated tapetum cells and enlarged microspores. **B**. Stage 10; tapetum cells are highly vacuolated and enlarged. Microspores commence degrading. **C**. Stage 11; microspores remain vacuolated, enlarged tapetum with reduced cytoplasm. **D**. Stage 12; degenerated tapetum and collapsed pollen grains. MSp, microspores; PG, pollen grains; Sm, septum; T, tapetum. Scale bars = 25 μm in **A**, scale bars = 50 μm in **B**, **C** and **D**.

Four out of the twenty-four wild type lines transformed with *At80MD* exhibited 15-50% fertility (Figure 6D). The remaining lines remained partially (90%) or fully fertile. The transcript levels of *At80MD* were all significantly higher than that of the endogenous *AtMYB80* in all the selected lines (Figure 6F). The highest expression level of *At80MD* was obtained in line 13, which showed 15% fertility. The transcript levels of the endogenous *AtMYB80* were reduced in all the lines when compared with the wild-type level. Tapetum and pollen development in the partially sterile *At80MD* lines was examined using light microscopy of anther sections. At stage 8, the tapetum cells were vacuolated and the microspores released from the tetrad were enlarged (hypertrophic) and irregularly shaped (Figure 7A). The tapetum cells became highly vacuolated and hypertrophic at stage 10. Microspore degradation had commenced and cellular debris was observed in anther locules (Figure 7B). At stage 11, a few pollen grains have developed normally in one anther locule and the tapetal layer is degenerating (Figure 7C). In a second locule, however, microspores and tapetum remained highly vacuolated and hypertrophic. Microspore debris was present and tapetal cell walls were intact. The cytoplasmic content of tapetal cells was greatly reduced. The tapetum had completely degenerated at stage 12. Pollen grains had collapsed and debris was attached to the endothecium layer (Figure 7D). The At80MD truncation protein may be able to compete with the endogenous AtMYB80 for binding the promoters of target genes, but fail to activate gene expression.

### Male sterility in Arabidopsis is induced by GhMYB80/BnMYB80-EAR fusion repressors

**Figure 8:**
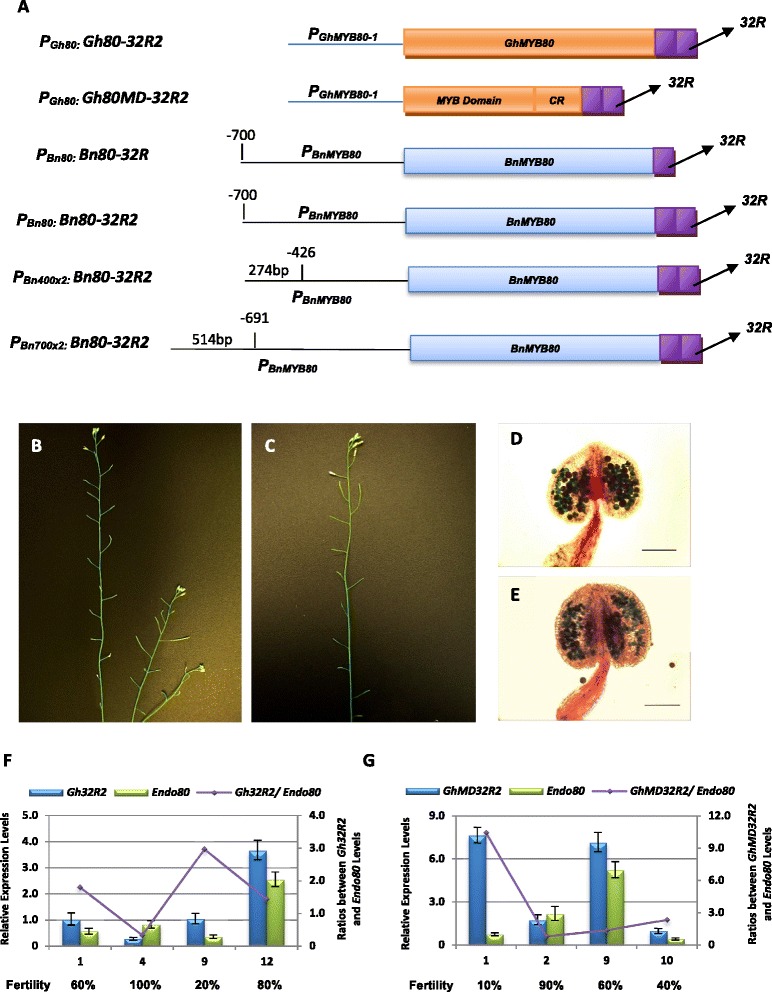
**Phenotype of silique elongation and expressional analyses of transgenes in the** ***P***_***Gh80***_ ***:Gh80-32R2*** **and** ***P***_***Gh80***_ ***:Gh80MD-32R2*** **Arabidopsis. A**. A schematic representation of the *P*_*Gh80*_ *:Gh80-32R2* and *P*_*Gh80*_ *:Gh80MD-32R2* chimeric constructs. **B** and **C**. Wild-type Arabidopsis possessing either the *P*_*Gh80*_ *:Gh80-32R2* **(B)** or *P*_*Gh80*_ *:Gh80MD-32R2* **(C)** transgene has a partially sterile phenotype. **D** and **E**. Alexander’s staining shows the majority of pollen grains lack cytoplasm and are aborted in the *P*_*Gh80*_ *:Gh80-32R2* **(D)** and *P*_*Gh80*_ *:Gh80MD-32R2* **(E)** anthers. Scale bar = 85 μm. **F** and **G**. The relative expression levels of the *P*_*Gh80*_ *:Gh80-32R2* **(F)** and *P*_*Gh80*_ *:Gh80MD-32R2* **(G)** transgenes in the selected lines. The higher the ratio (*P*_*Gh80*_ *:Gh80-32R2* or *P*_*Gh80*_ *:Gh80MD-32R2* vs. *AtMYB80*), the lower the plant fertility obtained. *Gh32R2*, *P*_*Gh80*_ *:Gh80-32R2*; *GhMD32R2*, *P*_*Gh80*_ *:Gh80MD-32R2*; *Endo80*, endogenous *AtMYB80*. Error bar represents SD.

Manipulation of AtMYB80 function has been employed to develop a reversible male sterility system in Arabidopsis. A chimeric construct of the full-length AtMYB80 with the SRDX EAR motif resulted in 60% of the transgenic lines exhibiting complete male sterility [2]. An EAR-like motif (LDLNLELRISPP), designated 32R, is a putative negative regulatory domain (NRD) found in AtMYB32 and shared by other MYB proteins in subgroup 4 [19,20]. We wished to determine if the GhMYB80 and BnMYB80 proteins are effective in inducing male sterility in Arabidopsis when the 32R motif is fused. In addition, to determine whether the effect is enhanced by truncating the MYB80 protein, adding two rather than one 32R motif or by increasing promoter strength. A full-length or a truncated *GhMYB80* was fused in frame with two copies of the *32R* sequence (*P*_*Gh80*_*:Gh80-32R2* and *P*_*Gh80*_*:Gh80MD-32R2*). The truncated sequence consisted of the MYB domain and the 44-amino acid region. Both chimeric constructs were driven by the 443bp *GhMYB80-1* promoter (Figure 8A). The full-length *BnMYB80* (C gene) coding sequence was also fused with one or two copies of the 32R EAR and placed under the control of a 700bp *BnMYB80* promoter (*P*_*Bn80*_*:Bn80-32R* and *P*_*Bn80*_*:Bn80-32R2*). The effect of double promoters was examined by using double 400 or 700bp *BnMYB80* 5’UTR sequences to drive the *BnMYB80-32R2* chimeric constructs (*P*_*Bn400x2*_*:Bn80-32R2* and *P*_*Bn700x2*_*:Bn80-32R2*) (Figure 8A).

PCR screening identified forty-one transgenic *P*_*Gh80*_*:Gh80-32R2* and sixty-three transgenic *P*_*Gh80*_*:Gh80MD-32R2* lines. Silique elongation in each line was examined. Approximately one-third of the transgenic *P*_*Gh80*_*:Gh80-32R2* lines and half of the *P*_*Gh80*_*:Gh80MD-32R2* lines showed less than 25% fertility (Figure 8B and C). A partially fertile phenotype (over 75% fertility) was observed in 34% of the transgenic *P*_*Gh80*_*:Gh80-32R2* lines and 3% of the transgenic *P*_*Gh80*_*:Gh80MD-32R2* lines, respectively (Additional file 7: Table. S4). Alexander’s staining of anthers from the severely sterile (less than 25% fertility) lines possessing either construct showed the majority of pollen grains lacked cytoplasmic content (Figure 8D and E). The expression levels of the *P*_*Gh80*_*:Gh80-32R2* and *P*_*Gh80*_*:Gh80MD-32R2* transgenes as well as the endogenous *AtMYB80* were examined in the selected lines using qRT-PCR. Plant fertility was shown to depend on the ratio between the transcript levels of the transgenes and endogenous *AtMYB80*. The higher the ratio (*P*_*Gh80*_*:Gh80-32R2* or *P*_*Gh80*_*:Gh80MD-32R2* vs. *AtMYB80*), the lower the plant fertility obtained (Figure 8F and G). The addition of two 32R copies to the 700 bp *BnMYB80* promoter driving *BnMYB80* (*P*_*Bn80*_*:Bn80-32R2*) was less effective than a single EAR sequence (*P*_*Bn80*_*:Bn80-32R*) (Table 1). Two copies of the 700 bp *BnMYB80* promoter driving the full-length *BnMYB80* gene (*P*_*Bn700x2*_*:Bn80-32R2*) were more effective than the two copies of the 400 bp *BnMYB80* promoter (*P*_*Bn400x2*_*:Bn80-32R2*). The BnMYB80-32R repressor induces male sterility more strongly in Arabidopsis than GhMYB80-32R when the two chimeric constructs were driven by their own promoters. The difference may reflect the shorter length (strength) of the *GhMYB80* promoter.

**Table 1:** **The number of the** ***P***_***Bn80***_ ***:Bn80-32R*** **,** ***P***_***Bn80***_ ***:Bn80-32R2*** **,** ***P***_***Bn400x2***_ ***:Bn80-32R2*** **and** ***P***_***Bn700x2***_ ***:Bn80-32R2*** **transgenic Arabidopsis lines obtained**

**Constructs**	**100% fertility**	**50-75% fertility**	**0% fertility**	**Percentage of the completely sterile lines**
*P*_*Bn80*_ *:Bn80-32R*	1	2	4	57%
*P*_*Bn80*_ *:Bn80-32R2*	2	5	3	30%
*P*_*Bn400x2*_ *:Bn80-32R2*	2	1	2	40%
*P*_*Bn700x2*_ *:Bn80-32R2*	5	7	17	59%

The percentages of the completely sterile lines carrying each construct are indicated.

## Discussion

### Comparison of MYB80 structure and function

Among the proteins encoded by the eight *MYB* genes cloned from Arabidopsis, Brassica and cotton, the MYB domain, an adjacent 44 amino acid sequence and an 18 amino acid C-terminal sequence are highly conserved. The latter is extended by eight amino acids in the two cotton proteins. A variable region of 131 to 139 amino acids is located between the 44-amino acid and the C-terminal sequences, sharing 10.7% identity between the eight MYB proteins.

The sequence conservation among MYB80 proteins suggests similar functions. *OsMYB80*, *TaMYB80*, *BnMYB80* (C gene) [6] and *GhMYB80*-*1* are all able to restore male fertility of the *atmyb80* mutant, implying functional conservation between monocots and dicots. The conserved 44 amino acid sequence is essential for MYB80 function as, when removed, the protein is unable to restore *atmyb80* fertility.

### *GhMYB80* in cotton anther development

The developmental stages of cotton anther development were found to closely resemble those of Arabidopsis (Additional file 7: Table S4). In Arabidopsis *AtMYB80* expression is strongest at stage 9 and tapetal cell degradation is initiated at stage 10 [1,4]. In cotton, however, tapetal cell degradation commences at anther development stage 9 and is largely completed at stage 10. These differences are consistent with the earlier increase (stage 7) and downregulation (stage 8) of *MYB80* transcript levels in cotton.

### Comparison of the *MYB80* promoters

The promoters of all eight *MYB* genes share an 80 bp sequence (approximately −300 to -380 bp upstream of the ATG) which includes four *cis*-elements, one of which is a MYB binding site. We have not yet ascertained the importance of these elements in driving gene expression. *GhMYB80* was more effective in restoring male fertility of the *atmyb80* mutant when driven by the *AtMYB80* (1105 bp) than the *GhMYB80* promoter (443 bp), presumably reflecting the difference in promoter length. This result implies that additional *cis*-elements driving expression are located in the −464 to −1105 region. Alternatively, the timing of *GhMYB80* promoter expression, which is perhaps slightly different from that of the *AtMYB80* promoter, may also reduce the effectiveness of *GhMYB80* promoter in complementing the *atmyb80* mutant. The reduced autoregulation caused by the ineffective *cis*-elements in *GhMYB80* promoter may contribute to the timing difference.

Two putative MYB-binding sites, namely MYB1 and MYB2, are situated -257bp and -246bp upstream of the transcription start site of the *AtMYB80* promoter. *GUS* expression appeared unaffected driven by the MYB1 mutated promoter. However, when both MYB elements were mutated, *GUS* expression no longer ceased at anther stage 10 in Arabidopsis, persisting into stage 12 in microspores and degraded tapetal cells. Although it is not clear yet whether a mutated MYB2 element alone would affect the *AtMYB80* expression, these results suggest MYB2 element plays a major role in the down-regulation of *MYB80* expression at the later stages. The two MYB *cis*-elements in the *AtMYB80* promoter are conserved in the promoter of the *B. napas MYB80* C gene and the two *GhMYB80* genes. However, they are absent from the wheat and rice *MYB80* gene promoters, suggesting *MYB80* downregulation may be regulated differently in monocots.

Disruption of the *AtMYB80* gene also changes the expression pattern of its promoter. Thus the *GUS* expression driven by the wild-type *AtMYB80* promoter was extended to stage 12 in the anthers of the homozygous *atmyb80* mutant. The expression levels of the truncated *AtMYB80* transcript were up-regulated in young *atmyb80* anthers as shown in the microarray and qRT-PCR analyses. These results suggest that AtMYB80 protein is involved in the negative auto-regulation of its expression at the later stages of anther development. AtMYB80 positively regulates the expression of some genes but represses the expression of others [3]. The mechanism by which MYB80 changes from an activator to a repressor is not known. Three other MYB proteins, AtMYB4, 7 and 32 possess an EAR-like sequence, and have been shown to repress their own promoters [20-22].

AtMYB80 positively regulates the expression of the aspartic protease encoding gene *UNDEAD.* A gene that must be downregulated if the correct timing of tapetal PCD is to be achieved [3]. Thus it is critical that *MYB80* expression is repressed at the appropriate stage of anther development. The downregulation of *GhMYB80* at late stage 8 in cotton anthers is consistent with the earlier tapetal degradation when compared with Arabidopsis.

### MYB80-EAR as an inducer of male sterility

The chimeric protein AtMYB80-EAR when introduced into Arabidopsis induces male sterility [2]. The GhMYB80 and BnMYB80 proteins fused with an EAR-like sequence, namely 32R, also resulted in male sterility in Arabidopsis. Since the sterility can be reversed [2] and MYB80 proteins from cotton, canola, wheat and rice have similar functions, the system provides a novel means to obtain hybrid vigour in crops. Important is the level of *MYB80-EAR* expression that can be achieved to ensure maximal levels of male sterility.

The transcript level of endogenous *AtMYB80* is reduced in all lines over-expressing *At80MD*. The overexpressed truncated protein may compete for the AtMYB80-interacting proteins, leading to the reduced expression of the endogenous *AtMYB80* gene. Whilst RNAi silencing of the endogenous *AtMYB80* in *At80MD* lines could not be excluded as responsible for the reduction in male fertility, the silencing does not appear to significantly affect the expression of the transgene *At80MD*.

When the *32R* sequence was fused with the truncated *GhMYB80MD* sequence and transformed into wild type Arabidopsis plants, the percentage of male sterile plants obtained was higher than when the full length *GhMYB80* sequence was used. Fifty percent of the *P*_*Gh80*_*:Gh80MD-32R2* lines were more than 75% infertile while the figure was 30% for the full length *P*_*Gh80*_*:Gh80-32R2* lines. The *At80*-*EAR* (*P*_*At80*_*:At80-SRDX*) construct resulted in 60% of Arabidopsis lines isolated exhibiting complete male sterility and silique abortion [2] whereas with *At80MD*-*EAR* (*P*_*At80*_*:At80MD-SRDX*) the figure rose to 75% [6].

A strong promoter is required to drive the MYB80-EAR construct to maximize the level of male sterility obtained. The 700bp *BnMYB80* promoter was more effective than the 400bp promoter, although two copies of the 700bp *BnMYB80* promoter were no better than a single copy. A single EAR sequence fused to the MYB80 protein was more effective than a double sequence. The *P*_*Bn700x2*_*:Bn80-32R2* (EAR x2) construct resulted in approximately 60% of lines being completely male sterile. However, a similar percentage of lines displaying complete male sterility was obtained when *Bn80-32R* (single copy of EAR) was driven by a single copy of the *BnMYB80* promoter (Table 1).

The results indicate that a combination of a strong promoter (driving tapetum and microspore expression) and a single copy of the EAR sequence fused to the MYB80MD protein will induce high levels of complete male sterility. In addition, the 32R EAR is less effective than the SRDX when fused to the MYB80 protein. This variability suggests the possibility of designing new EAR sequences with even greater repressive activity.

## Conclusions

In this paper we extend our studies on *MYB80* genes to include the Brassica A and C genomes and the two cotton orthologs. Promoter and functional analysis of the orthologs found that the expression pattern and function of a cotton ortholog are conserved and that *MYB80* expression is negatively autoregulated. The developmental stages of the cotton anther were examined and *GhMYB80* expression found to cease prior to the commencement of tapetal degradation.

The conservation of *MYB80* genes in crops is of interest as manipulation of the gene’s expression provides a novel reversible male sterility system for obtaining hybrid vigour. We examined ways to optimize inhibition of *AtMYB80* expression using a chimeric MYB80 fused with the EAR sequence from AtMYB32. A single EAR copy fused to the truncated MYB80 driven by a strong promoter (for example, *B.napus MYB80*) proved to be the most efficient construct for obtaining male sterility.

## Methods

### Plant materials and transformation

Wild type canola (*B. napus cv*., Westar), brown mustard (*B. juncea*) and cotton (*G. hirsutum*, Coker 315) seeds were obtained from Division of Plant Industry, Commonwealth Scientific and Industrial Research Organisation, Canberra, Australia. Wild type brussel sprout (*B. oleracea*) is an Australian commercial variety. Wild type *Arabidopsis thaliana* accession Columbia (Col-0) and the *atmyb80* T-DNA insertion mutant lines were obtained from GABI-Kat (Max Planck Institute for Plant Breeding Research), the European Arabidopsis Stock Centre. Arabidopsis, canola, brown mustard and brussel sprout were grown in a plant growth room at 22°C under constant illumination. Wild type cotton was grown in a glasshouse with a temperature of 30°C/22°C (day/night). Arabidopsis transformation was performed using *Agrobacterium tumefaciens* strain GV3101 by dripping approximately 50 μL of the infiltration medium (2-day-grown Agrobacteria culture, 5% sucrose, 0.03% Silwet) onto each floret. The dripping procedure was repeated once a week for three weeks. Constructs were transformed into the wild type or fertile heterozygous *atmyb80* plants. Genotypic and phenotypic analysis of the segregating populations was then performed in the T1 generation.

### Plasmid construction

The coding sequences of the *GhMYB80-1/-2, BnMYB80A*, *BjMYB80A*, and *BoMYB80C* were generated by PCR amplification using primers designed from the conserved DNA sequences. The *GhMYB80-1/-2* promoter sequences were obtained by the genomic walking method using the BD GenomeWalker kit (Clontech) according to the manufacturer’s protocol.

The *GhMYB80* and *BnMYB80* promoter fragments were cloned into pENTR/D-TOPO vector (Life Technologies) and then transferred into pKGWFS7 or pGWB533 destination vector using the LR clonase reaction. DNA fragments of the *P*_*Gh80*_*/P*_*At80*_*:Gh80*, *GhMYB80*-*EAR*, and *AtMYB80* truncation constructs were cloned into pGWB501 destination vector. Four serial deletion of the *AtMYB80* promoter fragments were amplified from the pPG construct [1] and cloned into the pBI101.1 vector using the restriction sites BamHI and HindIII. Site-specific mutagenesis was carried out using the Muta-Gene Phagemid kit (Bio-Rad) according to the manufacturer’s protocol. The two mutated promoters were cloned into the pBI101.1 vector. The double *BnMYB80* promoters were created by fusing two 400 or 700 promoter repeats. The 5’ promoter repeats contain the sequence immediately upstream from TATA box (excluding the TATA box), generating a 274 plus 426 bp (double 400 promoter) and a 514 plus 691 bp (double 700 promoter) sequences. The *BnMYB80*-*EAR* fragments were fused with the single or double *BnMYB80* promoter and then cloned into pCAMBIA1380 binary vector (CAMBIA). Gene specific primers are listed in Additional file 8: Table S5.

### Floral buds measurement and RT/qRT-PCR Analysis

The length (from the tip of the bud to the base of the petiole) and width (the longest horizontal dimension from one side to another side) of cotton floral buds were measured under a microscope. Half of the anthers from each bud were embedded for semi-thin sectioning. The second half was used for RNA extraction. Measurements and RNA extraction were replicated for each size. Arabidopsis anther stages were determined according to the length of Arabidopsis flower bud [23].

Total RNA was extracted from the isolated anthers or floral buds using the RNeasy plant kit (Qiagen). The first strand of cDNA was synthesized using SuperScript™ III Reverse transcriptase (Life Technologies, Catalog # 18080–044) according to the original protocol. Eliminating genomic DNA contamination was then performed by DNase digestion (Life Technologies, Catalog # 18068–015). The conditions for RT-PCR amplification of cDNA were as follows: 94°C for 3 min; 26 to 28 cycles of 94°C for 30 s; 55-60°C for 30 s and 72°C for 40 s; one cycle at 72°C for 7 min. RT-qPCR was performed using the SensiFAST SYBR & Fluorescein Kit (Bioline, Catalog # BIO-96020) on the MyiQ iCycler (BIO-RAD). The PCR conditions were as follows: 94°C for 3 min; forty cycles of 94°C for 30 s; 55-60°C for 30 s; 72°C for 20 s; one cycle at 72°C for 5 min. Data was analysed using the iQ5 (BIO-RAD) software. Relative gene expression level was calculated using the primer efficiency^^(−deltaCT)^ method. Fold change was calculated using the primer efficiency^^(−delta deltaCT)^ method. The Arabidopsis *UBIQUITIN10* (*UBQ10*) and *G. hirsutum UBIQUITIN1* (*UBI1*) genes were used as reference. Gene specific primers are listed in Additional file 8: Table S5.

### Sectioning of resin-embedded floral buds

Arabidopsis florets were fixed, embedded, and sectioned as described by Li [2]. Cotton anthers dissected from floral buds were fixed in FAA fixation (50% ethanol, 5% acetic acid, 3.7% formaldehyde, 41.3% water) and then embedded in LR White. Sections of cotton anther were performed in the same way as the Arabidopsis florets.

### Histochemical assay of transformed arabidopsis plants

Fresh Arabidopsis floral buds were prefixed in 1% glutaraldehyde solution (made up in 50 mM sodium phosphate buffer, pH 7.4) and then covered with X-gluc solution (0.5 mg/ml X-gluc in dimethylformamide, 50 mM sodium phosphate buffer, and 0.05% Triton X-100). Samples were incubated at 37°C for 4–16 hours and washed with 95% ethanol to remove the chlorophyll. GUS activity was examined under a dissecting microscope. Arabidopsis anthers were stained with Alexander’s stain [24] and examined microscopically.

### Availability of supporting data

The data set of DNA sequences supporting the results of this article is available in the GenBank repository, accession numbers KM675703 – KM675707.

## Additional files

Additional file 1: Figure S1. Nucleotide sequence alignment of MYB80 homologs from Arabidopsis (*AtMYB80*), canola (*BnMYB80A* and *BnMYB80C*) and cotton (*GhMYB80-1*). Additional file 2: Table S1. Summary of GUS activities in the transgenic lines possessing the *AtMYB80* promoter-*GUS* deletion constructs. Additional file 3: Figure S3. Partial promoter sequences alignment of the *MYB80* homologs from Arabidopsis (*pAt80*), canola (*pBn80A* and *pBn80C*), cotton (*pGh80*), wheat (*pTa80*) and rice (*pOs80*). Additional file 4: Table S2. GUS activities in the *atmyb80* mutant lines possessing the *AtMYB80* promoter-*GUS* construct. Additional file 5: Table S3. Comparison of anther development stages (3 to 11) between Arabidopsis and *G. hirsutum*. Additional file 6: Figure S2. Expression analyses of *GhMYB80* in *G. hirsutum* anther using semi-quantitative RT-PCR. Additional file 7: Table S4. Plant fertility (percentage of the elongated siliques versus the total siliques) and number of the *P*_*Gh80*_ *:Gh80-32R2* and *P*_*Gh80*_ *:Gh80MD-32R2* transgenic lines. Additional file 8: Table S5. Primer sequences used in this article.

## Abbreviations

CMS: Cytoplasmic male sterility
EAR: ERF-associated amphiphilic repression
GUS: β-glucuronidase
PCD: Programmed cell death
qRT-PCR: Quantitative reverse transcription-PCR
