# Supplementary material for: MYB80 homologues in Arabidopsis, cotton and Brassica: regulation and functional conservation in tapetal and pollen development
Source: BMC Plant Biol. 2014 Oct 14;14:278. doi: 10.1186/s12870-014-0278-3 (PMC4205283; doi:10.1186/s12870-014-0278-3)
Supplement: Additional file 1: Figure S1. — Nucleotide sequence alignment of MYB80 homologs from Arabidopsis (AtMYB80), canola (BnMYB80A and BnMYB80C) and cotton (GhMYB80-1). [file 12870_2014_278_MOESM1_ESM.pdf]

```
 AtMYB80     (1) -ATCGATATTCAAGTTTGTAATAATGTGGATTTACCACTACTTGTTTACAACAAAAGATG
BnMYB80A     (1) ------------------------------------------------------------
BnMYB80C     (1) ---------------------------------------------------T-CACGTGG
GhMYB80-1    (1) AAAACCCATTTTAACTGTAATAAATCTTAATGGAAAAGTATTTCGTAACAA--AACGATA

 AtMYB80    (60) ACACAAGTAGGGTCCTTCGTGATTTAGAACAAAAGGTACAAGTT--CGTACGCGAAAAAG
BnMYB80A     (1) -------------------GAATTCAGATTAGGAA-TAC--GTT--CGTACGGGGTAGGG
BnMYB80C     (9) GGGTCCTGTAATTGACTCGGAAGTCTCAAACGTAAATGC--GTT--CGTGCGCGAAAGAG
GhMYB80-1   (59) ACGTGACTAAAATGTAACATTTCAAACATAAGTGGCTAAAATTTAACTTGAGGCAAACAA

 AtMYB80   (118) GTACTTCTACAA-GATTCTGTGCTTTTCACGAATGCTCTCATAGTCTC-ACTAATGCACA
BnMYB80A    (37) A-----CGAAAG-GAAAGGGTACATGTCTT-GAGATTTTTTGTGTAACAACGACTGCTCA
BnMYB80C    (65) A-----CAA----GAAACGGTACTTGTATAAGATTCTCTCTGTGTTTCCATGAATGCACA
GhMYB80-1  (119) AAATGACTATTTTGATAGTTTACCCTTTTTTTACCGTTAAAAATTGATCCTTATATATC

 AtMYB80   (176) CGAACGCTTTCGCAATATTCTCCGTACTTGTCGGTA----CTTCACGAATACATTCCCTT
BnMYB80A    (90) TTAATGCGCC--ACAAGTACGCCCT-------CGAA----TCTCAT-AATATTCTCCGTA
BnMYB80C   (116) CGTACGCCCTCGAGACATTCTCCAT-------GCGT----CACCAA-GATATT-TCCTTA
GhMYB80-1  (179) AGCATTAGGT--ATACGTAGCACATCATATGTGGTTATTCCGTCAGTCACATT-AATTTT

 AtMYB80   (232) ACAAAATGAAAAAAAA--GTTGTCATAACTTTTTTAAAAAAATCGTGTCATAACTTCAAT
BnMYB80A   (136) CATCAC-GAAGACATA-CATTCTCTTACAAAAAGGACAAGGACATTGTCA-AACATCAGT
BnMYB80C   (163) GAAAAAAGAATATGCATCATAAACTCGAGTTTAATACTTTGTCAAAAAAA-AACTCCAGT
GhMYB80-1  (236) TAATAGTAAAAATGGATGAAGTGAACAATTTTTTCTTTAATTTAATGTACATTGACTAAT

 AtMYB80   (290) ATGATCACAAATTAT--ATTAAAAAAGAGTACAATTTCACTTCATAATCCAAAGGCTAGT
BnMYB80A   (193) GT---CACAG-TTAT--ATAAATCTA---TAAGAGCTCAACTTATAT-------------
BnMYB80C   (222) CT---AACAA-TTAT--ATAAACCTA---TAATATCTCAATTTATATATAATGATTCAAC
GhMYB80-1  (296) TTG--CACATTTTATGAATAAAAAGACAATACAATTT-AACTCTTAATATATGAGTCACC

 AtMYB80   (348) TTGTTATCCAAGTCGTTCTACCAATCCCCAAAATGTTTAAAAGTTGGAGTTTGCATAGAG
BnMYB80A   (231) ---TCAT-------GTT-TA--AATG----------------------------------
BnMYB80C   (273) CCCTAAT-------GTT-AA--ACTGCCAAAACTTATTGT--TTGCGTAGAT-AATATTT
GhMYB80-1  (353) GCGCTACTTTCACCCAAAAAATAGTACGTGGGGTGATT-TAA--GACAAGGC-CACACGT

 AtMYB80   (408) AATATCAGAAGAATTATATAATCTAAGTGTAAATATATGA--AGAATATAATTTTATGGG
BnMYB80A   (244) -------TAAGTAATTTATTTT-TGGGTGAAATTCGATAA-TACGATTTGAAAAAAG---
BnMYB80C   (320) GACAGAATAATTTATTTATGTT-TGGGTGAAATTCGATAAATACAATATGAAAAAAAGAA
GhMYB80-1  (409) GGAAGTTGAAACATTGGAAGGGCTGTAAGTAA-TGTATGA-TGAAATCTGAAAGAATG--

 AtMYB80   (466) TGAATAATCTGAATGTAAAAGGAGTTGACCAGGCGTTGAGTGGGTGACCTACCGGCTTAA
BnMYB80A   (292) TG--------GGATATACAAGGAGTTGACCAAGCGTAGGGTGGGTGACCTACCTGCCAAA
BnMYB80C   (379) TGATGAAAATGGATATACAAGGAGTTGACCAAGCGTAGAGTGGGTGAACTACCTGCCAAA
GhMYB80-1  (465) TCA--------AAGAAGAGAAGAGTTGACCGGGCGTCTTGAGGGTGAGCTACCGGCCAAT

 AtMYB80   (526) GGGAATCACACGCTCTGTAGCGTTGAATTCTTTAAAGAGTTTAATCAA--TTCATGCA--
BnMYB80A   (344) GGGAATCACACGCTCTGTAGCGTTGAATTCTTTATAGAGTTTAATCAC--TTCATGCA--
BnMYB80C   (439) GGGAATCACACGCTCTGTAGCGTTGAATTCTTTACAGAGTTTAATCAC--TTCATGCA--
GhMYB80-1  (517) CC--TTCACACACTCAACAGCGTTGAATTCTTTAATAAACTCATGCACATTTGATGGATG

 AtMYB80   (582) -CATCTGATGCTAATTTTTACTATGTTTTCATTTTTGTTAAATCAATTTTATTATATATA
BnMYB80A   (400) -CATTTGATGCTAATTTCATC---GTTTTAATTCTT-------------TTATAAG---TA
BnMYB80C   (495) -CATTTGATGCTACTTTCATC---GTTTCAATTTTTGT----------TTATTATA--TA
GhMYB80-1  (575) TCTTTTCATGCATATATGCAATATCTTTCCCCCTTTGTATATAA----ATAATATACCTT

 AtMYB80   (641) TACATACATTTCCTTTCTTCTTTTGCAATTTAAAAG----------CCTTGATTACAAGA
BnMYB80A   (441) TGCATACATTTTCTTTCTTCAGTTTCAATTTAATAG----------TCTCGATTACAAGA
BnMYB80C   (539) TATACACATTTTCTTTCTTCCTTTACAATTTAAAAG----------CCACGATTACAAGA
GhMYB80-1  (631) AACAAATAAAATGTACACCCCCAAACATTTCTCACTTCCCCCCGCCCCTTTTTTTCTTTA

 AtMYB80   (691) AAGTACAAAAATCAAAAGTTGGCAG--TTTTAATTCTTTGGGTGGGGCAATCTTTGATAT
BnMYB80A   (491) AAACAAAGAAATTAAAGTTTGGTAG--TTTTGATTCTTTTGG----GCAATCTTTGATTT
BnMYB80C   (589) AAACAAAGAAATTAAAGTTTGGCAG--TTTTAATTCTTTTAG----GCAATCTTTGATTT
GhMYB80-1  (691) TTTCGACCTCATCAAAGTTTTTTAAGCTATAGCTAGCTGTAGCACAAGAAGCACTCAGCC

 AtMYB80   (749) ATATAAGTTTTTTTCATTTCTTAACCAAATTTTCTTTTTTCTCGTTAG--TAAATTAAGT
BnMYB80A   (545) ATCTC----TTTTTCATCTCTTCACCAAATTTTCTTTTGTTTCTTGTGGCTGAATTAACT
BnMYB80C   (643) ATCTC----TTTTT------TTC------TTT-CTTGTGATCAGTA------AATTAATT
GhMYB80-1  (751) ATTTGC---TTTTTCTTTTCCTTTTAATTTTTCCCATTTGTTGCCGAG--TCTTTTAACC

 AtMYB80   (807) AAAGAGTAATCAAATCGATCAAGAGATAAAAACTAGAAAGAAAGAAAGAAGAAATGGGTC
BnMYB80A   (601) AAAGAAAAATCGATTTGTTTTCGAGATAAAAACTAGAAAAGAAAAGAAAAGA-ATGGGTC
BnMYB80C   (680) AAAGAGAGA-CGATCGA------GAGATAAAAACTAGAAAG--------AAAGA-ATGGGTA
GhMYB80-1  (806) TTATTTTTAGCATTTAATTT--GTCATGGAA--TAAAAGTGTAATAAAAAAAAATGGGTC

 AtMYB80   (867) GGATTCCATGTTGTGAAAGGAGAATGTGAAGAGAGGACAATGGACTCCTGAAGAAGACA
BnMYB80A   (660) GGATTCCATGTTGTGAAAGGAGAATGTGAAAAGAGGACAATGGACTCCTGAAGAAGACA
BnMYB80C   (726) GGATTCCATGCTGTGAAAGGAGAATGTGAAGAGAGGGCAATGGACTCCTGAAGAAGACA
```

```
GhMYB80-1   (862)  GGATTCCATGTTGTGAGAAAGACAATGTTAAAAGAGGACAGTGGACACCCGAGGAAGACA

  AtMYB80   (927)  ACAAATTGGCTTCTTATATTGCTCAACATGGTACTCGTAATTGGCGTCTCATCCCTAAGA
 BnMYB80A   (720)  ACAAATTGGCTTCTTACATTGCTCAACACGGTACTCGTAATTGGCGTCTCATCCCTAAAA
 BnMYB80C   (786)  ACAAACTGGCTTCTTACATTGCTCAACATGGTACTCGTAATTGGCGTCTCATCCCTAAAA
GhMYB80-1   (922)  ACAAGCTCTCTTCTTACATTGCCCAACATGGCACCCGCAATTGGCGTCTCATCCCCAAGA

  AtMYB80   (987)  ATGCTGGTAATTTAAATTTTCTCTATATTTTTTTTAATG-------TTTTAT--------
 BnMYB80A   (780)  ACGCTGGTAAAATTAATTTGCACT-TCTTTAATCAAGTAGGAATAGTCTTATAAAGATCT
 BnMYB80C   (846)  ACGCTGGTATATTTAATTTACACT-TTTTTC-TAAAATA---------TTAT--------
GhMYB80-1   (982)  ATGCTGGTCTGTGT--TTTGTTCT-TATCAC-CCTAATA-------TCCAATG-------

  AtMYB80  (1032)  ---------TTAGTCAAGTGATCA--------------------------------TTGT
 BnMYB80A   (839)  GATTATCCATCACTCTGCTGAATATTTCAAGACAAACTTTTAAAACAAACAAATGTTTGT
 BnMYB80C   (887)  -----TTCTTTAGTCTAGT-AATAT--------------------------AATGAT-GT
GhMYB80-1  (1024)  ------CCATTGGATTGCAATGCA-------------------------------ATTGT

  AtMYB80  (1051)  -----------------AATGTAATATA---ATGAATCGACAAAATTTGGT------C--
 BnMYB80A   (899)  AAAGGTAAAGATTTGGTTTTATAATGTACATACATATAAATGAG--CTGGTGAAA-ATTT
 BnMYB80C   (914)  ---GGTCTA-ATTTG-TTTGAGCGTGTATTAATGGATCGGTGAAACCTGGT------TGT
GhMYB80-1  (1047)  ----------GTTATTTTTTGTTGTTTT---AAAAGTAAAAGAAATTGGGTTGGGGATGA

  AtMYB80  (1083)  -TTTAATAGGGTTGCAAAGATGTGGGAAGAGTTGTAGGCTGCGATGGACAAACTATCTGC
 BnMYB80A   (956)  GTTTTGTAGGATTGCAGAGATGTGGGAAGAGTTGTAGACTAAGATGGACGAACTATTTGC
 BnMYB80C   (963)  TATATATAGGATTGCAGAGATGTGGAAAGAGTTGTAGACTACGGTGGACAAACTATTTGC
GhMYB80-1  (1094)  TTTTGCTAGGTCTCCAAAGATGTGGGAAAAGCTGCCGATTGCGATGGACTAATTACCTTC

  AtMYB80  (1142)  GTCCGGATTTGAAACATGGCCAGTTCTCGGAGGCTGAAGAACATATCATTGTCAAGTTTC
 BnMYB80A  (1016)  GTCCTGACCTGAAACACGGACAGTTTTCAGACGCTGAAGAACATATCATTGTCAAGTTTC
 BnMYB80C  (1023)  GTCCTGACCTGAAACATGGTCAATTTTCTGAGGCTGAAGAACATATCATCGTCAAGTTTC
GhMYB80-1  (1154)  GGCCGGACCTTAAACATGGCCAGTTCTCCAATGCTGAGGAGCAAACTATTGTGAAGCTCC

  AtMYB80  (1202)  ACTCTGTTCTTGGTAACCGGTATGATCACTACTCACTAGATCTAATTAACTACGGTTTTA
 BnMYB80A  (1076)  ACTCTGTTCTTGGTAACAGGTATGACTCTTAAATACAATCCTCTCTTAAGAATCAATAAA
 BnMYB80C  (1083)  ACTCTGTTCTTGGTAACCGGTATGACTTCTTTATA--------TGTTATGTACCA-----
GhMYB80-1  (1214)  ATTCTGTTGTTGGCAACCGGTA--------------------------------------

  AtMYB80  (1262)  TGATTTTCTTGAAAAGATTTAATCTAGGTGTATAAATTACAGATGGACTCATAAATATCG
 BnMYB80A  (1136)  ATTTCGTTACGTCA----TTTCTTGGGATGTGAAAATTATGGATAGACTAA--AGTGGCC
 BnMYB80C  (1130)  -------TACCTCA----TTAC---GGCCTTCAAACTTTTC-ATACATTCA--ATTTTCT
GhMYB80-1  (1236)  ------------------------------------------------------------

  AtMYB80  (1322)  GGTTTCTTTACTAAGTTTTTGA-AATTTTGATGCT-GACTTACTGATGAATTGTTTTTAT
 BnMYB80A  (1190)  TGTTTTAATAATAAATTATTATGCAATATGATAGTCTCCAGACTGTTCAAAAAAGAACGT
 BnMYB80C  (1173)  TGAATCCAT--TTCTTTGGGGATATTTAAGAAAGT-TACAGATAGACTCAAACAGGGCGG
GhMYB80-1  (1236)  -------------------------------------ACAAATCGCTAAACAATG-----

  AtMYB80  (1380)  GAGTTTTTGACAAGTTTCTTGGGACACAAGAAACTGAATAAG-------------ACATA
 BnMYB80A  (1250)  GATGAGAATCTCACAAGTTCTTGGGAACAAGAAACTGAATATCGATCTAACTTTAGCAGG
 BnMYB80C  (1230)  GGTTTGT-TCTTATTCTGTTTTGG-------ATTTTGATTAT--ATC--ATTTGTCCACA
GhMYB80-1  (1254)  G--------------CTTCCTTAG---------------------------------ACA

  AtMYB80  (1428)  AGTTT-TGTTCTATATAC-------CATAATACCAAATCTAAGTAT--A------GGG-T
 BnMYB80A  (1310)  AATTGCTGTTATATATACTTAACTACAGAGTTTAATTTTTGAGTCTAAAATGAATTGCTT
 BnMYB80C  (1278)  AGTTTCT-TGGGAAACAAGAAAC---AGAA--TAAGACGTAAGCCT---------TGCTT
GhMYB80-1  (1267)  AGACT-----------A----------G---ATAAAATGCCACCCT-------------A

  AtMYB80  (1471)  TTTAAACTTTTGGGAACTT-----TTA-ATTTTCTATA--AATTTGTTTTCGACTCATAA
 BnMYB80A  (1370)  CTCTAACACTATTTGACTCAGAAACTAAAGGTTGTAATCAAAGTTCCAAAAAAAACAAAA
 BnMYB80C  (1323)  TATATACCAGGTCTAACTC------TAGAT-TTCTAAT---ATTTCGAGAAGTTTCTTCA
GhMYB80-1  (1290)  TTTCAAGC--TT----C--------------TTTTGTT----------------------

  AtMYB80  (1523)  A---TAGAGTTTGAT-GATGTATTGCTGATGAATAGTCTGGGATT-TTTCATGAGAAACA
 BnMYB80A  (1430)  AATCTAAAGGTTGAAAGATTGCT-GATGATA--TTATATGTTTTTATTTCCAAAAGAGCA
 BnMYB80C  (1373)  ----TAAGTTTTGAAATATATATAGCTGATC--GT---TGGATTTATTTTCCAACGAGCA
GhMYB80-1  (1308)  --------GTTTTAT---TA----GCTCAT----------GTTTT-----------GGGA

  AtMYB80  (1578)  ACAGTTAACATCTTTCTTG------------------------GG---AAACAT----TT
 BnMYB80A  (1487)  ATAGTTAGCAAAATTTCTGAGGAACAAGAAAGTGAATAAGAAAGACATAAACCTTGCTTA
 BnMYB80C  (1424)  ATAGTTAGCAAGAATT-----------------GAATAA----GACATCAACCTTATTTT
GhMYB80-1  (1332)  TTAGCTATTAACTTTG------------------------------------TC----TA

  AtMYB80  (1607)  AATAATCTTTTGACATATA----TGGTTCAGGTGGTCGTTGATTGCGGCGCAACTTCCTG
 BnMYB80A  (1547)  AGTTTTCTTTTGACGTATATGCATGGTTCAGGTGGTCGTTGATTGCGGCGCAGCTTCCAG
 BnMYB80C  (1463)  AATTTTCTTTTGACCTATA----TGGTTCAGGTGGTCGTTGATTGCGGCCCAGCTTCCTG
GhMYB80-1  (1352)  A--A----TTTGTACCACA------TTGCAGCTGGTCCTTGATTGCAGCACAGTTACCTG

  AtMYB80  (1663)  GTCGGACAGACAACGATGTGAAAAATTATTGGAACACGAAGCTGAAGAAGAAGTTGTCAG
 BnMYB80A  (1607)  GTCGAACAGACAACGATGTGAAAAACTATTGGAACACGAAGCTGAAGAAGAAGTTGTCGG
 BnMYB80C  (1519)  GTCGAACAGACAACGATGTGAAAAATTATTGGAACACAAAGCTGAAGAAGAAGTTGTCGG
GhMYB80-1  (1400)  GTCGGACAGACAATGATGTTAAAAATCATTGGAACACCAAGCTGAAGAAGAAGCTTTCAG
```

```
  AtMYB80   (1723) GAATGGGAATAGATCCGGTGACCCACAAGCCTTTCTCGCATCTAATGGCAGAGATCACCA
 BnMYB80A   (1667) GAATGGGGATAGATCCAGTTACTCACAAGCCTTTCTCGCACCTAATGGCAGAGATCACCA
 BnMYB80C   (1579) GAATGGGAATAGATCCCGTAACCCACAAGCCTTTCTCGCATCTAATGGCAGAGATAACCA
GhMYB80-1   (1460) GCATGGGTATCGATCCTGTGACACACAAGCCTTTCTCTCACCTCATGGCTGAGATAGCCA

  AtMYB80   (1783) CTACACTTAATCCTCCTCAGGTTTCTCACCTAGCCGAAGCTGCCCTCGGCTGTTTCAAGG
 BnMYB80A   (1727) CTACACTCAACCCTCCCCAGGTCTCTCACCTCGCTGAAGCTGCACTCGGTTGTTTCAAGG
 BnMYB80C   (1639) CTACACTCAATCCTCCTCAAGTCTCACACCTCGCTGAAGCTGCCCTCGGATGTTTCAAGG
GhMYB80-1   (1520) CTACATTGGCACCACCGCAGGTGGCTCATTTAGCTGAAGCGGCACTAGGGTGTTTCAAGG

  AtMYB80   (1843) ACGAGATGCTTCACTTGCTCACCAAGAAACGTGTTGACCTAAACCAAATCAACTTTTCAA
 BnMYB80A   (1787) ACGAGATGCTTCACTTGCTCACCAAGAAACGTGTTGACCTAAACCAAATCAACTTCTCAA
 BnMYB80C   (1699) ACGAGATGCTTCACTTGCTCACCAAGAAACGTGTTGATCTAAACCAAATCAACTTCTCCA
GhMYB80-1   (1580) ATGAAATGCTCCACCTGCTAACTAAGAAACGTATCGATTTCCAGCT--TCAACAATCAAA

  AtMYB80   (1903) ACCATAACCCT------AACCCAAACAACTTTCACGAGATT--GCTGATAATGA------
 BnMYB80A   (1847) GCCCTAACCATAACCATAACCCTAACAACTTTAACCAAACT--GTTGATAACGA------
 BnMYB80C   (1759) GCCCTAACC------------CTAACAACTTTACCCGAACC--GTTGATAGCGA------
GhMYB80-1   (1638) TCCGGGACAAGGGAATAATACCACAGTTCCTTACAGCAAACAAGATGAGAAAGATGATAC

  AtMYB80   (1949) AGCTGGTAAGATAAAGATGGATGGTTTGGACCATGGGAATGGGATAATGA-AGTTATGGG
 BnMYB80A   (1899) AGCTGGTAAGATGAAACTGGATT---------ATGGTAATGGGATAATGA-AGCTATGGG
 BnMYB80C   (1799) AGCTGGTAAGATGAAAATGGATGGTTTGGAGAATGGTAATGGGATAATGA-AGCTATGGG
GhMYB80-1   (1698) AGTTGAGAAGATCAAACTGAATTTAT----CAAGGGCTATACAAGAACCAGACATGCTTC

  AtMYB80   (2008) ACATGGGTAATGGATTCTCATATGGATCCTCTTCGTCTTCGTTTGGGAATGAAGAAAGAA
 BnMYB80A   (1949) ACATGGGTAATGGATTCTCGTATGGATCATCTTCCTCGTCCTTTGGGAATGATGAAAGGA
 BnMYB80C   (1858) ACATGGGGAATGGATTCTCCTATGGATCTTCTTCGTCATCGTTTGGGAATGAAGACAAAA
GhMYB80-1   (1754) CCTTGAATAAACCATGGGAGTCTACTAGTACAAGAGCAACATCGGCTAATTTTGAAGGGG

  AtMYB80   (2068) ATGATGGATCAGCGTCTCCTGCCGTTGCAGCTTGGAGGGGTCACGGAGGAATA-CGTACC
 BnMYB80A   (2009) ACGAGGGATCCGCGTCTCCTGCGGTTGCGGCGTGGAGGGGTCACGGTGGAATA-CGTACA
 BnMYB80C   (1918) ATGATGGAGCTGCGTCTCCTGCGGTTGCGGCGTGGAGGGGTCACGGTGGAATA-CGTACA
GhMYB80-1   (1814) GTTGTGGTGTTTTCCCTACATCTGTGACAGGATATCATCATTATGGCCCATCATCTTTTG

  AtMYB80   (2127) GCGGTAGCTGAAACCGCGGCAGCGGAGGAGGAGG--AGAGAAGGAA-GCTGAAGGGAGAA
 BnMYB80A   (2068) TCAGTGGCTGAGACGGCGGCAGTGGAGGAGGAAG--AGAGGAGGAA-GCTGAAGGGAGAA
 BnMYB80C   (1977) GCGGTGGCTGAAACTGCGGCAGCGGAGGAGGAAG--AGAGGAGGAA-ATTGAAGGGAGAA
GhMYB80-1   (1874) CCAATGAAGGGGGCGGTTCCGGCTCACCATGGAGCCAGAGTATGTGTACGGGAAGCACAT

  AtMYB80   (2184) GTG-----GTTGATCAAG--AGG--AGATTGGATCTGAA-GGAGGAAGAGGAGATGGAAT
 BnMYB80A   (2125) GTG-----ATGGAACAAG--AGG--AGATTGGATCTGAA-GGAGGAAGAGGAGATGGAAT
 BnMYB80C   (2034) GTG-----GTGGACCAAG--AGG--AGAATGGATCTCAA-GGAGGAAGAGGAGATGGAAT
GhMYB80-1   (1934) GTACCGCAGGGGAACAAGTTAGGTCACATGAGAAATTAAAGGATGAAAATGGTGAAGAAT

  AtMYB80   (2234) GACGATGATGAGGAACCA------TCATCATCATCAACATGTGTTTAATGTGGATAATGT
 BnMYB80A   (2175) G---ATGATGAGGAGGCAACATGATCAACATCAGCAACATGCGTTTAATGTGGATAACGA
 BnMYB80C   (2084) GTTGATGATGAGGAGCCAGCATGATCAACATCAACATCATGTGTTTAATGTGGACAATGT
GhMYB80-1   (1994) TTC-AAGGTGGGAAAGAA--ATTAAGAATGCAACAAGCATA--TTCAATACAGATTGTGT

  AtMYB80   (2288) CTTGTGGGATTTACAAGCTGATGATCTCATCAATCATATGGTTTGA--------------
 BnMYB80A   (2232) CTTGTGGGATTTACAGGCTGATGATCTCATTAATCATATGGTTTGA--------------
 BnMYB80C   (2144) CTTGTGGGATTTACAAGCTGATGATCTCATTAATCATGTGGTTTGA--------------
GhMYB80-1   (2049) GTTATGGGATATACCATCTGATGATCTTATCAACCCTATTTATAGAGAAGCCTTTAACAA

  AtMYB80   (2334) ----------
 BnMYB80A   (2278) ----------
 BnMYB80C   (2190) ----------
GhMYB80-1   (2109) CAAAAAATAA
```

Supplementary Figure S1. Nucleotide sequence alignment of MYB80 homologs from Arabidopsis (*AtMYB80*), canola (*BnMYB80A* and *BnMYB80C*) and cotton (*GhMYB80-1*). Yellow highlight represents the conserved nucleotides. Blue or green highlight represents the partially conserved nucleotides. The conserved *cis*-elements in the promoters are boxed, including the W-box (TTGAC), MYB element (A/TACC), GTGANTG10 element (TCAC), DOFCOREZM binding site (A/TAAAG). The putative two MYB bind sites targeted for mutagenesis are underlined. The translation start and stop codon are in bold letters.
