# Supplementary material for: MYB80 homologues in Arabidopsis, cotton and Brassica: regulation and functional conservation in tapetal and pollen development
Source: BMC Plant Biol. 2014 Oct 14;14:278. doi: 10.1186/s12870-014-0278-3 (PMC4205283; doi:10.1186/s12870-014-0278-3)
Supplement: Additional file 2: Table S1. — Summary of GUS activities in the transgenic lines possessing the AtMYB80 promoter-GUS deletion constructs. [file 12870_2014_278_MOESM2_ESM.pdf]

Supplementary Table S1. Summary of GUS activities in the transgenic lines possessing the *AtMYB80* promoter-*GUS* deletion constructs.

Construct	Line number	GUS expression
pPG	1	strong
	2	strong
	3	moderate
	4	moderate
1651-pBI101.1	1	strong
	2	moderate
	3	moderate
	4	strong
	5	moderate
	6	moderate
284-pBI101.1	1	weak
	2	moderate
	3	weak
	4	weak
	5	weak
	6	weak
256-pBI101.1	1	very weak
	2	very weak
	3	very weak
240-pBI101.1	1	not detected
	2	not detected
	3	not detected
	4	not detected
