# Supplementary material for: MYB80 homologues in Arabidopsis, cotton and Brassica: regulation and functional conservation in tapetal and pollen development
Source: BMC Plant Biol. 2014 Oct 14;14:278. doi: 10.1186/s12870-014-0278-3 (PMC4205283; doi:10.1186/s12870-014-0278-3)
Supplement: Additional file 3: Figure S3. — Partial promoter sequences alignment of the MYB80 homologs from Arabidopsis (pAt80), canola (pBn80A and pBn80C), cotton (pGh80), wheat (pTa80) and rice (pOs80). [file 12870_2014_278_MOESM3_ESM.pdf]

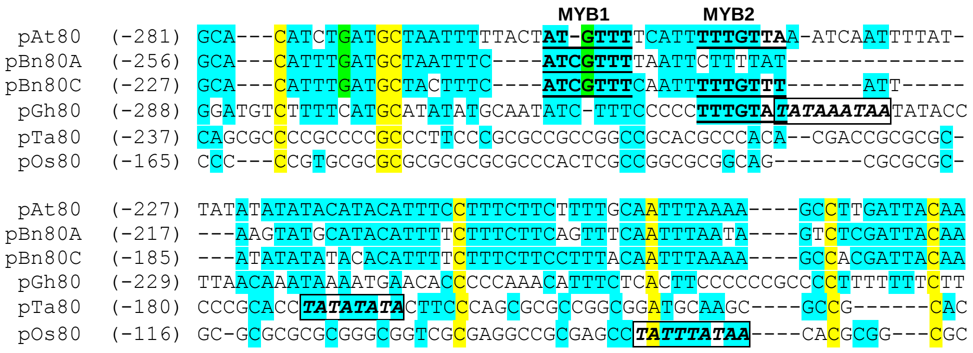

Supplementary Figure S3. Partial promoter sequences alignment of the *MYB80* homologs from Arabidopsis (*pAt80*), canola (*pBn80A* and *pBn80C*), cotton (*pGh80*), wheat (*pTa80*) and rice (*pOs80*). The putative two MYB binding sites are underlined and the putative TATA boxes are indicated.
