# Supplementary material for: MYB80 homologues in Arabidopsis, cotton and Brassica: regulation and functional conservation in tapetal and pollen development
Source: BMC Plant Biol. 2014 Oct 14;14:278. doi: 10.1186/s12870-014-0278-3 (PMC4205283; doi:10.1186/s12870-014-0278-3)
Supplement: Additional file 4: Table S2. — GUS activities in the atmyb80 mutant lines possessing the AtMYB80 promoter-GUS construct. [file 12870_2014_278_MOESM4_ESM.pdf]

Supplementary Table S2. GUS activities in the *atmyb80* mutant lines possessing the *AtMYB80* promoter-*GUS* construct. Heter, heterozygous *atmyb80* mutant; Homo, homozygous *atmyb80* mutant.

Line number	*atmyb80* mutant	GUS activities in anther by developmental stages
2	Heter	5 - 9
4	Heter	5 - 9
5	Heter	5 - 9
6	Heter	5 - 9
7	Heter	5 - 9
8	Heter	5 - 9
9	Heter	5 - 9
13	Heter	5 - 9
10	Homo	5 – 13
14	Homo	5 – 13
