# Supplementary material for: MYB80 homologues in Arabidopsis, cotton and Brassica: regulation and functional conservation in tapetal and pollen development
Source: BMC Plant Biol. 2014 Oct 14;14:278. doi: 10.1186/s12870-014-0278-3 (PMC4205283; doi:10.1186/s12870-014-0278-3)
Supplement: Additional file 5: Table S3. — Comparison of anther development stages (3 to 11) between Arabidopsis and G. hirsutum. [file 12870_2014_278_MOESM5_ESM.pdf]

Supplementary Table S3. Comparison of anther development stages (3 to 11) between Arabidopsis and *G. hirsutum*. Sizes (length and width) of the selected *G. hirsutum* floral buds corresponding to each anther developmental stage are indicated.

**Stages**	**Arabidopsis** (Sanders *et al.*, 1999)	***G. hirsutum***	***G. hirsutum* floral bud sizes**	
			**Length (mm)**	**Width (mm)**
3	The primary parietal and sporogenous layers generate the secondary parietal layers and sporogenous cells, respectively.	The secondary parietal layers and cells are apparent.	4.2 – 4.4	2.3 – 2.4
4	Four-lobed anther pattern with two developing stomium regions generated. Vascular region initiated.	Formation of the epidermis, endothecium, middle layer and tapetum has been initiated. Vascular region appears.	4.4 – 4.8	3.2 – 3.5
5	Four defined locules esTablelelished. Microspore mother cells appear.	The microspore mother cells appear.	6.4 – 6.5	4.1 – 4.2
6	Microspore mother cells enter meiosis. Middle layer is crushed and degenerates. Tapetum becomes vacuolated.	Microspore mother cells commence meiosis and the tapetal cells become vacuolated.	7.3 – 7.6	4.3 – 4.5
7	Meiosis completed. Tetrads of microspores free within each locule. Remnants of middle layer present.	The tapetal cytoplasm is condensed and tetrads appear in the anther locules.	8.6 – 8.8	5.3 – 5.6
8	Callose wall surrounding tetrads degenerates and individual microspores released.	Microspores are released from the tetrads. The tapetal cell walls have been degraded.	9.7 – 9.9	6.2 – 6.4
9	Microspores generate an exine wall and become vacuolated.	**Degeneration of tapetal layer appears to commence.** Microspores are vacuolated.	10.5 – 10.6	6.8 – 7.1
10	**Tapetum degeneration initiates.** Less vacuolated microspores exist.	The tapetum has been degraded. Remnants of tapetal cells visible. The microspores are still vacuolated.	12.4 – 12.8	7.5 – 7.8
11	Pollen mitotic divisions occur. Tapetum and septum cell degeneration initiates. Stomium differentiation begins.	Early pollen grains appear.	17.1 – 17.5	10.4 – 10.8
