# Supplementary material for: MYB80 homologues in Arabidopsis, cotton and Brassica: regulation and functional conservation in tapetal and pollen development
Source: BMC Plant Biol. 2014 Oct 14;14:278. doi: 10.1186/s12870-014-0278-3 (PMC4205283; doi:10.1186/s12870-014-0278-3)
Supplement: Additional file 6: Figure S2. — Expression analyses of GhMYB80 in G. hirsutum anther using semi-quantitative RT-PCR. [file 12870_2014_278_MOESM6_ESM.pdf]

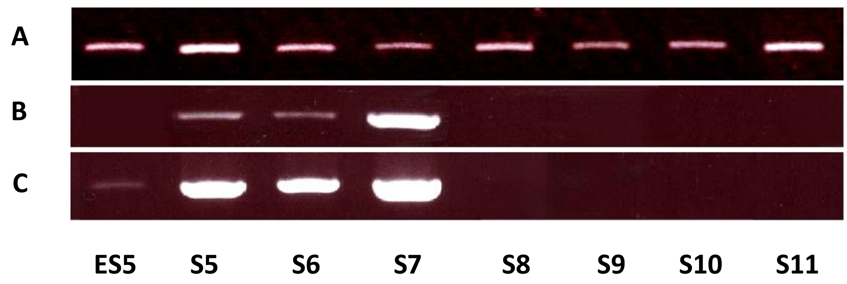

Supplementary Figure S2. Expression analyses of *GhMYB80* in *G. hirsutum* anther using semi-quantitative RT-PCR. A. Transcript of the *G. hirsutum β-tubulin* reference gene (26 cycles). B. Transcript of *GhMYB80* at 26 cycles. C. Transcript of *GhMYB80* at 28 cycles. ES5, early stage 5; S5 to S11, stages 5 to 11.
