# Supplementary material for: MYB80 homologues in Arabidopsis, cotton and Brassica: regulation and functional conservation in tapetal and pollen development
Source: BMC Plant Biol. 2014 Oct 14;14:278. doi: 10.1186/s12870-014-0278-3 (PMC4205283; doi:10.1186/s12870-014-0278-3)
Supplement: Additional file 7: Table S4. — Plant fertility (percentage of the elongated siliques versus the total siliques) and number of the P Gh80 :Gh80-32R2 and P Gh80 :Gh80MD-32R2 transgenic lines. [file 12870_2014_278_MOESM7_ESM.pdf]

Supplementary Table S4. Plant fertility (percentage of the elongated siliques versus the total siliques) and number of the P_{Gh80}:*Gh80-32R2* and P_{Gh80}:*Gh80MD-32R2* transgenic lines.

Plant fertility (%)	P_{Gh80}:*Gh80-32R2*	P_{Gh80}:*Gh80MD-32R2*
0	0	0
<25	12	30
25-75	15	31
>75	14	2
