# Supplementary material for: MYB80 homologues in Arabidopsis, cotton and Brassica: regulation and functional conservation in tapetal and pollen development
Source: BMC Plant Biol. 2014 Oct 14;14:278. doi: 10.1186/s12870-014-0278-3 (PMC4205283; doi:10.1186/s12870-014-0278-3)
Supplement: Additional file 8: Table S5. — Primer sequences used in this article. [file 12870_2014_278_MOESM8_ESM.pdf]

Supplementary Table S5. Primer sequences used in this article. Nucleotide sequences are in the 5’ to 3’ order.

Name	Nucleotide sequence
**Gene cloning**	
Br80F1	GCGTTTCTTGATGCGTGCTA
Br80F2	AGCCTAAAAGGCTAGAGTTC
Br80F3	AGGAATACGTTCGTACG
Br80F4	GGATATACAAGGAGTTGACCA
Br80R1	TCAAACCATATGATTAATGAGATCATCAGC
Br80R2	CATTCTTTTCTTTTCTTTTTCTAGTTTTTATCTCGA
Gh80PF1	CACCAAAACCCATTTTAACTGTAATAAATC
Gh80PF3	CACCAGTTGAAACATTGGAAGGGTT
Gh80SR2	GCCTACATTATTATTTTTTGTTGTTAAAGGCTT
**Vector construction**	
At80-1651 F	AGCAAGCTTCTTCTCCTTCTTCTCGGGATTACTC
At80-1105 F	AGCAAGCTTGGGCAAGGAGCTTCTATGGCCAA
At80-284 F	ACGAAGCTTATGCACATCTGATGCTAATTTTTA
At80-256 F	ATTAAGCTTTGTTTTCATTTTTGTTAAATCAAT
At80-240 F	AGCAAGCTTAAATCAATTTTATTATATATATAC
103+35 R	CGAGGATCCAGGAGTCCATTGTCCTCTCTTC
M1-1	CATCTGATGCTAATTTTTACTATTTTTTCATTTTTGTTAAATCAATTT
M2-2	CATCTGATGCTAATTTTTACTATTTTTTCATTTTTTTTAAATCAATTTTAT TATATATAT
At80P-TF	CACCGGCAAGGAGCTTCTATGGCCAA
At80P-Gh	CTTTCTCACAACATGGAATCCGACCCATTTCTTCTTTCTTTCTTTCTAG
Gh80-F1	ATGGGTCGGATTCCATGTTGTGAG
Gh32R	TTAAGGAGGACTTATTCTTAGCTCCAGATTTAAGTCCAAACCTGGTGGA CTGATCCTGAGTTCAAGGTTCAAATCTAATCCTTTTTTGTTGTTAAAGGC TTCTC
GhMD32R	TTAAGGAGGACTTATTCTTAGCTCCAGATTTAAGTCCAAACCTGGTGGA CTGATCCTGAGTTCAAGGTTCAAATCTAATCCATCGATACGTTTCTTAG TTAGCAGGTGG
At80MP-F	GATCCGGTGACCCACAAGCCTCTAAACCAAATCAACTTTTCAAACC
At80MP-R	AGGCTTGTGGGTCACCGGATC
At80MD-R	TCAGTCAACACGTTTCTTGGTGAGCAA
Bn80-F1	ATGGATCCTCACGTGGGGGTCCTGTAATTGAC
Bn80-F2	ATGGATCCGAAGTCTCAAACGTAAATGCGT
Bn80-F3	ATGGATCCTGATTCAACCCCTAATGTTAAACTG
Bn80-R1	TCTTTCTTTCTAGTTTTTATCTCTC
Bn80-32R	CAGTAAGCTTTTAAGGAGGACTTATTCTTAGCTCCAGATTTAAGTCCAA ACCAACCACATGATTAATGAGATC
Bn80-32R2	TGACAAGCTTTTAAGGAGGACTTATTCTTAGCTCCAGATTTAAGTCCAA ACCTGGTGGACTGATCCTGAGTTCAAGGTTCAAATCTAATCCAACCACA TGATTAATGAGATC
**RT-PCR/qPCR**	
Gh80-F1	ATGGGTCGGATTCCATGTTGTGAG
Gh80-R3	TGTAGTGGCTATCTCAGCCATGAGGTGAGA
qAt80-F	CTCCATCTTGTCCTTCGTTTG
qAt80-R	GGTCTGGCGGGATGCCTTCC
qGh80-F	TAATTACCTTCGGCCGGACC
qGh80-R	TCCGACCAGGTAACTGTGCT
GhTUB-F	CAGGTATGCCATTCTTTGGGAGG

GhTUB-R	CGGGATCCATTCAACAAAGTAAGA
GhUBQ-F	CTCCATCTTGTCCTTCGTTTG
GhUBQ-R	GGTCTGGCGGGATGCCTTCC
UBQ-F	TCCGGATCAGCAGAGGCTTA
UBQ-R	TCAGAACTCTCCACCTCAAG
At80En-F	GAGGAGGAGGAGAGAAGGAA
At80En-R	CATCAGCTTGTAAATCCCAC
At80SR1	CCAAATAAAAATCAAACCATATG
qMP-LV	CACAAGCCTCTAAACCAAATCAACTT
At80R2	ACCCATGTCCCATAACTTCATTATC
At80CNS-F	TTCTCGCATCTAATGGCAGAG
At80MD-RT	GCGCGCCCACCCTTTCAGTCAAC
